# Supplementary material for: Patterns of Intron Gain and Loss in Fungi
Source: PLoS Biol. 2004 Nov 30;2(12):e422. doi: 10.1371/journal.pbio.0020422 (PMC532390; doi:10.1371/journal.pbio.0020422)
Supplement: Table S1 — Also available at http://genes.mit.edu/NielsenEtAl/. (4.3 MB ZIP). [file pbio.0020422.st001.zip › NielsenEtAl/html/136.html]

AN8188.1.NCU07774.1.MG09458.1.FG05854.1


```
 CLUSTAL W (1.82) Multiple Sequence Alignments - Introns Inserted


Sequence 1: NCU07774.1	124 aa
Sequence 2: MG09458.1	125 aa
Sequence 3: FG05854.1	125 aa
Sequence 4: AN8188.1	150 aa
Alignment Length: 152 aa
Number Identitical Residues: 84 aa
Alignment Score (without introns) 3746


MG09458.1 	---------------------------MVIVKDIEIF~SMCEHHLVPFTGK0MHIGYIPA
NCU07774.1	---------------------------MVIVKDIEVF~SMCEHHLVPFTGK~MHIGYIPS
FG05854.1 	---------------------------MVIVKDIEVF0SLCEHHLVPFTGK0MHIGYIPN
AN8188.1  	MLYFTKGYEENVRDLVNGAVFHEDHDELVIVKDIDVF~SLCEHHMVPFTGK0MHIGYIPD
          	    :.. ...  .  ..:   .. ..:******::* *:****:****** ******* 

MG09458.1 	NSVIGLSKLPRIAELFARRLQIQERLTKEVANAIFEVLKPQGVAVVMESSHLCMVTRGVQ
NCU07774.1	NAVIGISKLPRIAELFARRLQIQERLTKEVANAIMEILKPQGVAVVMESSHLCMVMRGVQ
FG05854.1 	ETVIGLSKLPRIAEMFARRLQIQERLTKEVAHAIMEILKPQGVAVVMESSHLCMVMRGVE
AN8188.1  	RRVLGLSKLARLAEMFSRRLQVQERLTKQVALAISEVLKPRGVGVVMESSHLCMVMRGVQ
          	. *:*:***.*:**:*:****:******:** ** *:***:**.*********** ***:

MG09458.1 	KTTATTITSCVLGCFEGRERTRNEFLSLIGVNRH
NCU07774.1	KTTSSTITSCVLGCFESREKTRLEFLSLIGVNR-
FG05854.1 	KTTTSTITSCVLGCFERKSKTRNEFLNLIGINKR
AN8188.1  	KTSSSTTTSCMLGCMRSSAKTREEFLTLL--NRK
          	**:::* ***:***:.   :** ***.*:  *:
```
